# Evaluation of Bonding Quality with Advanced Nondestructive Testing (NDT) and Data Fusion †

**DOI:** 10.3390/s20185127

**Published:** 2020-09-08

**Authors:** Bengisu Yilmaz, Abdoulaye Ba, Elena Jasiuniene, Huu-Kien Bui, Gérard Berthiau

**Affiliations:** 1 Ultrasound Research Institute, Kaunas University of Technology, K. Barsausko St. 59, LT-51423 Kaunas, Lithuania; elena.jasiuniene@ktu.lt; 2 Institute of Research in Electric Power of Nantes Atlantique (IREENA), University of Nantes, 37 Boulevard de l’Université BP-406, CEDEX, 44602 Saint-Nazaire, France; abdoulaye.ba@etu.univ-nantes.fr (A.B.); huu-kien.bui@univ-nantes.fr (H.-K.B.); gerard.berthiau@univ-nantes.fr (G.B.); 3 Department of Electronics Engineering, Kaunas University of Technology, Studentu St. 48, LT-51367 Kaunas, Lithuania

**Keywords:** nondestructive testing (NDT), ultrasonics, induction thermography, adhesive bond, data fusion

## Abstract

This work aims to compare quantitatively different nondestructive testing (NDT) techniques and data fusion features for the evaluation of adhesive bonding quality. Adhesively bonded composite-epoxy single-lap joints have been investigated with advanced ultrasonic nondestructive testing and induction thermography. Bonded structures with artificial debonding defects in three different case studies have been investigated: debonding with release film inclusion, debonding with brass film-large, debonding with brass film-small. After completing preprocessing of the data for data fusion, the feature matrices, depending on the interface reflection peak-to-peak amplitude and the principal component analysis, have been extracted from ultrasonic and thermography inspection results, respectively. The obtained feature matrices have been used as the source in basic (average, difference, weighted average, Hadamard product) and statistical (Dempster–Shafer rule of combination) data fusion algorithms. The defect detection performances of advanced nondestructive testing techniques, in addition to data fusion algorithms have been evaluated quantitatively by receiver operating characteristics. In conclusion, it is shown that data fusion can increase the detectability of artificial debonding in single-lap joints.

## 1. Introduction

Given the rise in the stage of composite materials, new joining technologies such as adhesive bonding have gained popularity in the aerospace industry. Adhesively bonded structures have a high strength-to-weight ratio and can join dissimilar materials and complex geometries. Additionally, adhesive joints preserve the structural integrity and smooth surface of composites compared to mechanical fasteners like rivets—i.e., composite structures—might be damaged via fiber breakage during riveting. However, not being able to determine the inclusions in the adhesive–composite interface may lead to significant strength reduction as well as unexpected catastrophic failures. In order to expand the application of adhesive joints, the geometry, size and position of these inclusions have to be identified via nondestructive testing (NDT) techniques [1]. In our recent studies, interface defects in adhesive-bonded structures are inspected with ultrasonic and thermography nondestructive testing techniques [2,3].

Ultrasonic NDT had been used to investigate the adhesive bond quality with adherend integrity and interface quality evaluation, including disbond detection [4,5,6,7,8,9]. In addition, investigations to detect weak and kissing bonds have been performed in various studies [10,11,12]. Conventional longitudinal pulse-echo ultrasonic inspection as well as advanced measurement techniques, such as acoustic microscopy, air-coupled ultrasound, and guided waves, have been used to evaluate bonding quality [13,14,15,16,17,18,19]. Moreover, nonlinear behavior has been related to bonding quality with nonlinear ultrasonic NDT [20,21,22]. Ultrasonic NDT has advantages to detect and position defects since it is a directional technique. While the classical pulse-echo technique outperforms the through transmission technique by being a one-sided inspection, it requires structure to be coupled with specific substances such as water. Air-coupled ultrasonics overcome this limitation; however, the high impedance difference between air and structures causes a significant loss in signal amplitude [14]. On the other hand, guided wave inspections allow large specimens to be inspected in a short period of time; Lamb waves have been reported to be an effective technique to determine bonding quality [23,24]. However, the analysis of the results has a high level of complexity, and it is usually specimen-specific.

Additionally, active thermography is a promising NDT technique to investigate bonding quality [25,26,27]. Active thermography has advantages such as being very responsive, sensitive, noncontact, and suitable for automation; therefore, it is used to detect manufacturing defects within adhesive bonding [28]. Defect detection with traditional light-based active thermography is highly influenced by thermal diffusion and the anisotropy of the structures. In the case of conductive adherends such as CFRP, induction thermography can reduce this limitation [2,29]. Although induction thermography has many strengths and increased effectiveness by volumetric heating, the thickness of the structures and the complexity of the results limit its application [30].

Moreover, shearography can have a very high resolution and short response time; however, it is only effective in the case of surface and subsurface defects and requires high-stress solicitation [31]. It is reported that shearography is a suitable nondestructive testing technique to detect disbonding and subsurface defects in aluminum bonds [26]. Also, where possible, X-ray tomography can be used to investigate inner defects in bonded structures [27]. However, for composite-adhesive joints, similar diffraction coefficients and structures with a high level of aspect ratios might create limitations in this expensive NDT technique [32]. Recently, electromechanical impedance mismatching and an adhesion quality test with laser shock had been proposed to evaluate bonding quality [31,33,34]. While extended NDT for adhesive bonding is promising, these systems are expensive and costly to maintain.

The nondestructive evaluation of bonding quality is a challenging task because adhesive bonding is an interfacial phenomenon involving a thin layer of material, usually less than 10 microns [35]. Although adhesive bonding evaluations with different nondestructive testing techniques have been performed over the past decades, the challenges continue to rise to establish the ultimate reliable NDT technique [3]. Each NDT technique is limited to deliver a reliable evaluation of bonding quality due to its methodological and physical capabilities. Hence, we propose a combination of ultrasonic and induction thermography with feature-based data fusion.

Data fusion has been introduced to nondestructive testing and evaluation by Gros et al. and the research interest continues to rise [36,37]. While the detailed categorization of data fusion reveals the advantages for sensor applications [38], the survey on data fusion techniques for nondestructive evaluation also highlights numerous studies [39]. The application on concrete samples mostly used ground-penetrating radar (GPR), impact echo and ultrasonic testing as data fusion resources while deploying several data fusion algorithms such as fuzzy logic [40,41], artificial neural networks (ANN) [42], Hadamard, and the Dempster–Shafer rule of combination [43]. Considering the variety in nondestructive evaluation of composite structures, data fusion studies focused on several different combinations of NDT techniques. While Gusenbauer et al. [44] improved porosity determination in composites with X-ray tomography and interferometer; Cuadra et al. [45] monitored the damage in composites with acoustic emission, digital image correlation (DIC), and thermography. Cao et al. [46] employed convolutional neural networks (CNN) in order to improve lock-in thermography imaging. Specifically, Daryabor and Safizadeh [1] worked on the image fusion for ultrasonic and thermography nondestructive evaluation of epoxy patches between composite and aluminum structures. They compared several basic and complex fusion algorithms, namely minimum, maximum, average, principal component analysis, wavelet transformation and pyramid. 

This work focuses on the evaluation of bonding quality with the fusion of ultrasonic inspection and induction thermography data. Composite-adhesive single-lap joints containing three different artificial debonding defects were investigated by both ultrasonic NDT and induction thermography. Saved data had been preprocessed for data fusion. The feature matrices emphasizing the defect presence have been extracted from ultrasonic and thermography data. These feature matrices have been used as the source of data fusion algorithms. The data fusion algorithms have been evaluated with quantitative sensitivity analysis. In addition to the previous works that focused on data fusion with ultrasonic nondestructive testing techniques and thermography, this work investigates different types of defects in composite-adhesive bonds and utilizes information theory-based data fusion algorithms. Also, this work contributes to the quantitative bonding quality evaluation efforts with receiver operating characteristic curves and area-under-curve calculations.

## 2. Materials and Methods

### 2.1. Sample Description

**Figure 1:**
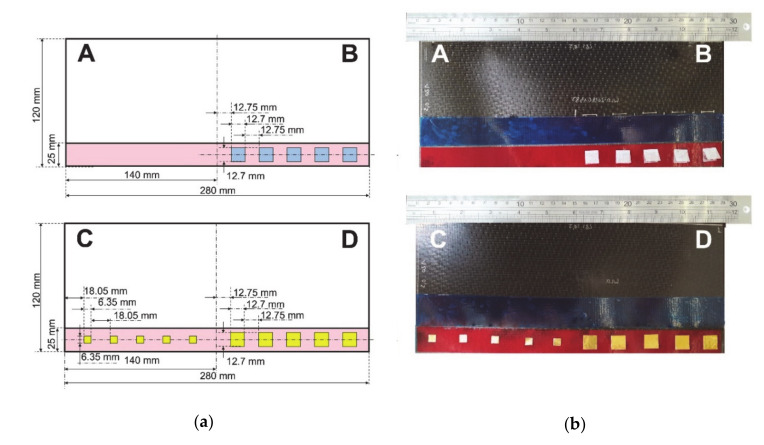
CFRP-epoxy single-lap joints with different bonding quality. (**a**) schematics and (**b**) pictures prior to bonding. (**A**: perfect bond, **B**: debonding with release film inclusion, **C**: Debonding with brass inclusion - small, **D**: Debonding with brass inclusion - large.).

Single-lap joints with carbon fiber-reinforced epoxy (CFRP) adherend and epoxy film adhesive were manufactured at COTESA, GmBH, Mittweida, Germany. Six layers of HexPly M21-5H satin woven prepreg, 2.22 mm thick, was used as an adherend. 3M Scotch-Weld AF163 k-red structural adhesive film epoxy with 0.24 mm theoretical thickness was used as adhesive. The epoxy film was placed on top of the cured CFRP adherends after required surface preparation. Single lap joints containing four different bonding quality were designed: three of them with interface inclusions, and one without any inclusion at pristine state as reference sample. Reference sample is called ‘perfect bond’ (Figure 1a(A) and Figure 1b(A)). 

As seen in Figure 1B five two-fold Wrigtlon 4600 (AirTech Europe, The City of Differdange, Luxemburg) release film inclusions with 12.7 mm edge length and 0.063 mm thickness were put on the bonding interface to demonstrate ‘debonding with release film’. Additionally, ‘debonding’ at the interface were represented with brass film inclusions. On the one part of the sample, five two-fold square brass films with 12.7 mm edge length and 0.05 mm thickness were inserted onto the interface (Figure 1D). On the other side of the sample, smaller square brass film inclusions with 6.35 mm edge length and 0.05 mm thickness were inserted (Figure 1C). Both defects might occur at the manufacturing stage of bonding structures due to foreign object inclusions, such as glove parts, cutting blade, etc.

### 2.2. Nondestructive Testing

Single-lap joint adhesive bonds with four different bonding quality have been investigated with two different nondestructive testing techniques: pulse-echo immersion ultrasonic NDT and transmission induction thermography.

#### 2.2.1. Ultrasonic Inspection

**Figure 2:**
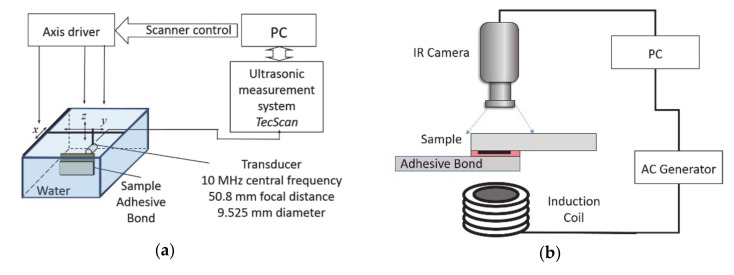
Experimental set-up schematics: (**a**) ultrasonic nondestructive testing (NDT) and (**b**) induction thermography.

Ultrasonic inspection was performed in water immersion tank with the pulse-echo technique. A single-element-focused transducer Olympus V375-SU (Olympus Scientific Solutions Americas Inc., Waltham, MA, USA) having 10 MHz central frequency, 9.525 mm diameter, and 50.8 mm focal distance was used. The single-lap joints were placed perpendicular to the transducer and the distance between the transducer and the sample was kept at 46.3 mm to place the focal point at the bonding interface. The inspections have been performed in whole bonding area for each single-lap joint (covering all five defects at the interface), and the step-size was 0.5 mm. For each measurement point, A-scans have been saved. Schematics of the experiment can be seen in Figure 2a.

#### 2.2.2. Induction Thermography

The single-lap joints with different bonding qualities have been investigated with induction (eddy current stimulated active) thermography. As described in an earlier study [3], the induction coil frequency plays a significant role in the experiment performance. Hence the design of the coil is selected as a helical coil inductor (inner diameter 15 mm, outer diameter 25 mm, height 30 mm, five turns, manufactured at IREENA institute, Saint-Nazaire, France) to achieve 105 kHz frequency. During experiments, the coil is excited with 200 Ampere power for 1 s. Starting from the excitation time, an infrared camera recorded the surface temperature for 60 s with a sampling frequency of 25 frames per second. The experiments have been performed in transmission mode, where the sample is placed in between the camera and coil. Schematics of the experiment can be seen in Figure 2b.

### 2.3. Feature-Based Data Fusion

Data fusion is a post-processing technique that uses a synthesis of the data collected by multiple sources (sensors or systems) in order to provide more accurate information. The literature defines the different levels of fusion as data-level, feature-level and decision-level [39].

**Figure 3:**
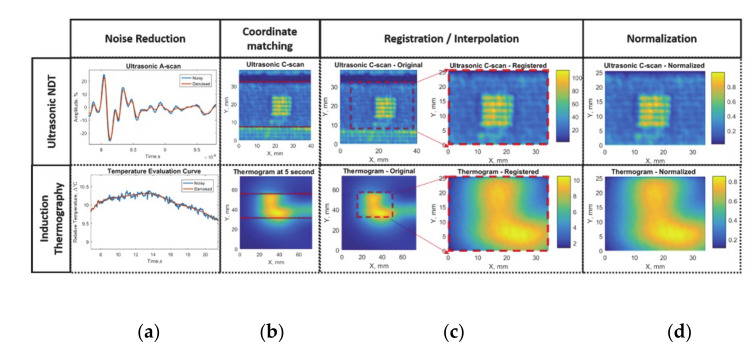
Data acquisition and preprocessing steps for feature-based data fusion: (**a**) noise reduction with digital filters, (**b**) coordinate matching according to bondline edge, (**c**) registration and interpolation of selected areas, (**d**) amplitude normalization.

Before the application of fusion algorithms, it should be guaranteed that the collected data is comparable. In this work, raw data gathered from NDT investigations have been preprocessed. A multi-step preprocessing approach has been followed as shown in Figure 3.

First of all, in order to eliminate error multiplication due to *noise*, data collected during ultrasonic inspection and induction thermography experiments have been filtered separately (Figure 3a). Since the experiments took place in different conditions, recorded data had local coordinate systems. However, to apply data fusion, the coordinates of each experiment should match the other. Therefore, data have been aligned according to the position of bonding edges to *match coordinates* (Figure 3b). While the point-by-point match was achieved by this operation, scaling of the data was performed to have the same coordinate system in both sets of data. Hence, the ultrasonic inspection data has been interpolated to match the same coordinate values in the *registration* step (Figure 3c). The data was re-centered, keeping the center of the defect at the midline of horizontal coordinates with the parallel top and bottom edges to the edge of the bondline. No further registration step needed due to the perpendicular position of the IR camera and ultrasonic transducer to the specimen. Last but not least, both ultrasonic and thermography data amplitudes have been normalized (0 to 1) (Figure 3d). 

**Figure 4:**
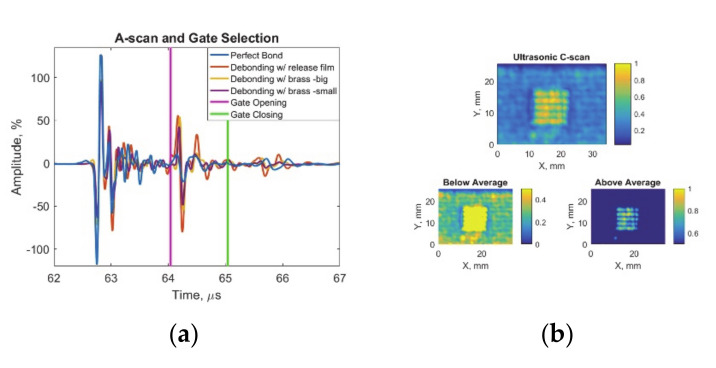
Ultrasonic response data with feature matrix image and decision procedures: (**a**) ultrasonic A-scan with gate at the interface reflection amplitude and (**b**) ultrasonic C-scan with below and above average for defect detection.

After preprocessing, features were determined by the known physical relationship between each technique and the samples. For the ultrasonic pulse-echo inspection, maximum amplitudes recorded at time of the interface reflection have been extracted as features. In order to visualize bonding quality at the interface, C-scan images have been created by peak-to-peak amplitude values inside the selected time gate. This gate has been determined according to the interface reflection time-of-flight calculations by the knowledge of thickness and acoustic wave velocity of adherend and adhesive. (Figure 4a).

The inclusions causing debonding defects at the interface reflected higher amplitude ultrasonic echoes to the transducer due to high impedance mismatch, whereas no-defect/pristine state of bonding transmitted most of the ultrasonic wave further due to similar impedance values. The normalized feature values were separated into two conditions (Figure 4b): no defect below the average value and a defect above the average value.

**Figure 5:**
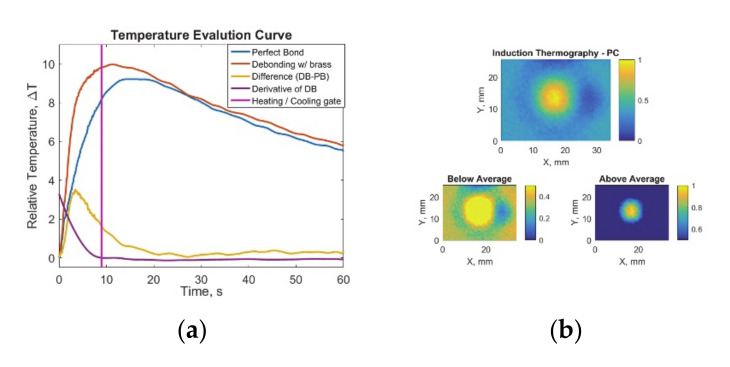
Induction thermography results with feature matrix image and decision procedures: (**a**) temperature evaluation curve with heating/cooling separation gate and (**b**) induction thermography -heating principal component analysis results with below and above average.

The induction thermography data have been evaluated with singular value decomposition-based principal component analysis (PCA). As described in the previous work [3], PCA allows to eliminate the nonuniform heating patterns and increase the defect contrast in thermography results. Since PCA calculates the eigenvectors within data and sorts them in ascending order; the first few principal components carry the most information [30]. To maximize defect detection, each recorded defected sample thermography data and perfect bonding sample thermography data was differentiated after alignment according to the bondline. As described in the previous study, the recorded surface temperature data was separated for heating part and cooling part with the novel separation algorithm, which depends on the constant rate of change (derivative) of the sample temperature to be reached (Figure 5a) [3].

Only for the heating part, PCA algorithm was applied with MATLAB software. While the first principal components have been neglected due to the nonuniform heating pattern, the second principal components were saved as features emphasizing the defect (Figure 5b). Due to the nature of defects (metal- and polyester-based) some would have higher values at the defected region and some would have lower values; therefore, the absolute values are considered before normalization. The defect is present at above-the average-values, whereas no defect is present on the below the average values (Figure 5b).

### 2.4. Data Fusion Algorithms

After preprocessing and feature extraction, six different fusion algorithms were applied to the compatible 2D feature matrices on the pixel level. The details of fusion algorithms are given in Table 1.

There are numerous data fusion algorithms that can be applied for two-dimensional feature-based fusion, such as basic combinations, wavelet-based combinations, artificial neural networks, Bayesian theory, and the Dempster–Shafer rule of combination [39]. In this study, a combination of basic and information theory-based fusion algorithms have been selected: average to indicate the equal performance of the sources, difference to clarify contradiction between sources, weighted average to highlight the importance of one source over the other, Hadamard to increase the signal-to-noise ratio, and Dempster–Shafer theory-based combination to highlight the importance of information theory.

As one of the basic fusion algorithms, *average* has been implemented. The resulting matrix has the average of each feature matrices. Then, to understand if two techniques are inversely correlated, *difference* algorithm has been performed. Afterward, two different *weighted average* algorithms—where one dataset is having four times higher importance than the other—have been studied (5UT-1TH, Table 1, Formula (3a)) where the weighted average of ultrasonic inspection matrix is four times higher than the thermography feature, (1UT-5TH, Table 1, Formula (3b)) where the weighted average of thermography feature is four times higher than the ultrasonic inspection). Furthermore, the *Hadamard product*, which is a simple algebraic operation based on pixel-wise multiplication of same-size matrices, has been applied to feature matrices [47]. The resulting matrix is a product of the pixel values on the same positions from different sources [43].

**Figure 6:**
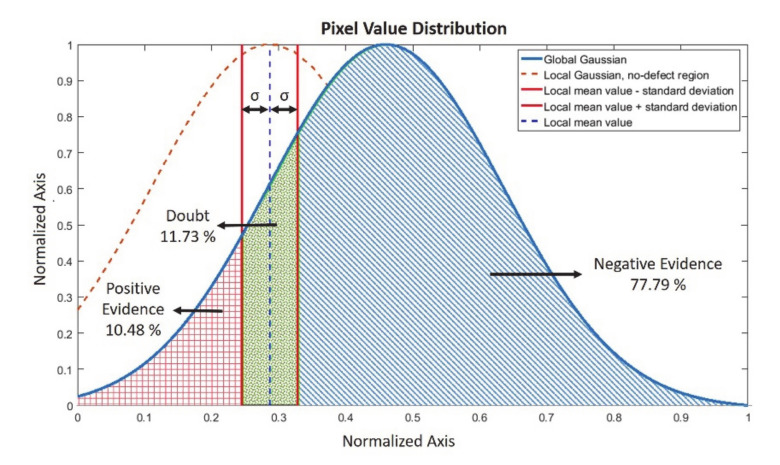
Dempster–Shafer belief percentage calculation for a random pixel and its local neighborhood on the no-defect region and global distribution for the induction thermography feature matrix.

Finally, the Dempster–Shafer (DS) rule of combination has been applied to the feature matrices. DS evidence theory is introduced by Shafer [48] as an expansion of Dempster’s theory [49]. In DS theory, the information from each source is considered as evidence of multiple events. The Dempster–Shafer rule of combination allows us to calculate a unique evidence mass (m) for a hypothesis by combining the evidence masses ($m_{1},m_{2}$); in other words, beliefs associated with this hypothesis by various sources or operators [36]. In our case, these hypotheses are defected (positive), not defected (negative) and unsure if it is defected or not (doubt). The combination ($m_{1}⊕m_{2}\left(A\right)$) has been calculated via the orthogonal sum of different hypotheses from different sources (Table 1, Formula (5)). The sources ($m_{1}\left(B\right)$, $m_{2}\left(C\right)$) are the feature matrices obtained from each NDT inspection. Where K represents the contradiction in the belief systems of two sources. If the K value is calculated close to 1, the calculated rule of combination results in very low values, and the rule of combination should be modified. Three hypotheses—positive, doubt, and negative—have been chosen according to the cross-section of local amplitude distribution over global Gaussian in the feature matrix. The local distribution has been calculated with the pixels and its surrounding (8) pixels’ values. In Figure 6, the Dempster–Shafer global distribution and belief percentage calculation for a random pixel and its local neighborhood on the no-defect region is presented.

According to the local distribution on the global Gaussian curve, the probability of three hypotheses have been calculated (Figure 6): positive evidence (DS-positive) where a defect is present corresponding to the left side of the Gaussian cross-section, negative evidence (DS-negative) where no defect is present, seen on the right side of the cross-section, and doubt (DS-doubt) where the plausibility is high, corresponding to the local amplitude variance crossing with the global Gaussian. For each pixel value, these three different belief probabilities have been calculated and then DS rule of combination algorithm has been applied. According to the graph in Figure 6, the selected point/pixel can be determined with a 77.79% probability that it is not in the defected region, with a 10.48% probability it is in the defected region and that it is a doubt with a 11.73% probability.

### 2.5. Evaluation of Different Techniques

The performance of each fusion algorithm and separate features have been evaluated quantitatively with Receiver Operating Characteristic (ROC) curves. In order to create ROC curves, each feature matrix and resulting fusion matrices have been analyzed for sensitivity and specificity. 

Firstly, according to the known position of the defect, an artificial reference matrix where defects have been chosen as 1 and sound area is chosen as zeros have been created. In other words, the knowledge on the position of the defects according to the known model helped to create a numerical example of defect/no-defect matrix, which was used for comparison with the other techniques and named as *real defect*. 

Every matrix has been binarized in order to evaluate it using ROC curves. For simplicity, histogram-based segmentation has been performed over a hundred (100) steps. According to the artificial reference matrix, each pixel segmented within the matrix has been classified with as true positive, false positive, true negative and false negative. –True Positive (TP) when there is defect in defect position, False Positive (FP) when there is defect in sound area, True Negative (TN) when there is no defect in sound area, False Negative (FN) when there is no defect in defect position-. According to sensitivity and specificity information [50] true positive rate (TPR) and false positive rate (FPR) have been calculated for each segmented matrix as follows, Equations (6) and (7):

TPR = TP / (TP +FN)(6)

FPR = FP / (FP+TN)(7)

Finally, the receiver operating characteristic curve has been obtained by plotting false positive rates against true positive rates. In order to quantitatively evaluate each fusion and feature result, area-under-curve (AUC) for each ROC curve has been calculated via trapezoids.

## 3. Results

The samples have been investigated with the above-described methodology. The results from perfect bond -no defect case- have been only used in order to eliminate environmental and system-based errors in induction thermography results. In this section, three different bonding quality investigations have been reported: debonding with release film inclusions (12.7 mm edge size), debonding with brass inclusion—Large (12.7 mm edge size), debonding with brass inclusion—Small (6.35 mm edge size) (Figure 1).

### 3.1. Case 1: Debonding with Release Film Inclusions (12.7 mm Edge Size)

The adhesive bond containing debonding with release film inclusion had been inspected using ultrasonic immersion NDT and induction thermography. Feature matrices have been obtained according to above-described post-processing methods (see Section 2.3). Data fusion algorithms (Section 2.4) have been applied to the feature matrices.

**Figure 7:**
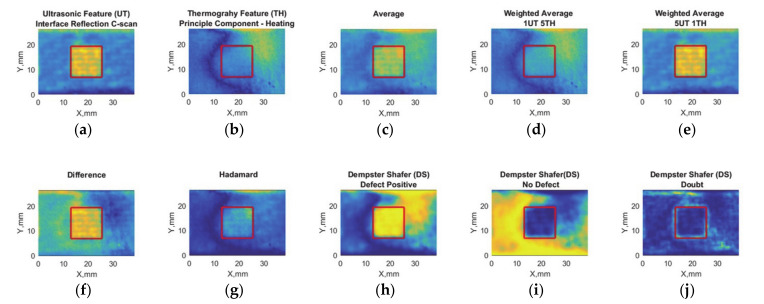
Debonding with release film (12.7 mm edge size) feature-based data fusion algorithm results: (**a**) ultrasonic feature (UT) with maximum values at interface reflection C-scan, (**b**) thermography feature (TH) principal component analysis response, (**c**) average of UT and TH, (**d**) weighted average where TH is five times more than UT, (**e**) weighted average where UT is 5 times more than TH, (**f**) absolute difference, (**g**) Hadamard fusion, (**h**) Dempster–Shafer fusion for defect placement, (**i**) Dempster–Shafer fusion for no defect positions, and (**j**) Dempster–Shafer fusion where doubt is high. The real position of the defect has been indicated with red squares.

The feature matrices for ultrasonic inspection and induction thermography with data fusion results are presented in Figure 7.

The real defect position has been shown with a red square. The ultrasonic feature indicates the defect region with a higher amplitude response than the sound area (Figure 7a). In the induction thermography feature matrix, the defect position does not have a high contrast compared to the sound area (Figure 7b). On the other hand, averaging data fusion results show smoothen feature (Figure 7c), while the defect contrast is much higher in weighted average 5UT-1TH (Figure 7e) than the others. The difference fusion matrix shows relatively high performance on defect detection (Figure 7f). On the other hand, Hadamard fusion indicates very low performance in defect detection (Figure 7g) while DS performs well in the defected region (Figure 7h) but not well in the sound area (Figure 7i).

**Figure 8:**
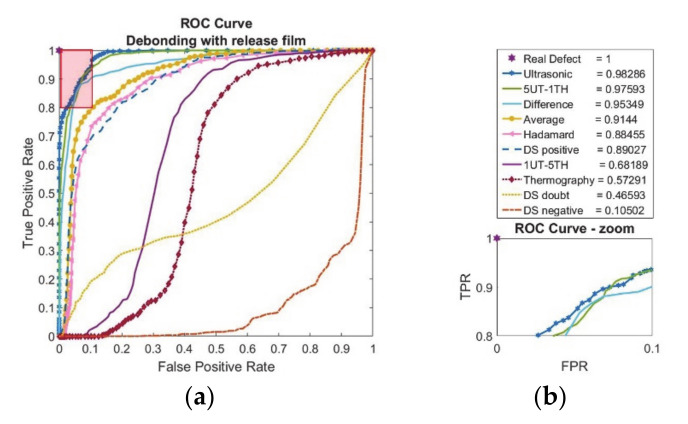
Debonding with release film (12.7 mm edge size) feature-based data fusion evaluation results with (**a**) receiver operating characteristic curve (ROC) and (**b**) area-under-curve (AUC) calculations with zoom image of ROC curve.

The receiver operating characteristic curves for adhesive bonds containing debonding with release film have been shown in Figure 8.

As seen in Figure 8b, the area under ROC curves have been calculated and indicated with the label. The best performance point is shown with ‘star’ at the position [0,1] where real defect is observed. According to Figure 8a, the ultrasonic feature and weighted average 5UT-1TH performs best. Furthermore, fusion results with difference, average, Hadamard, and DS-positive seem to perform reasonably well. The area-under-curve (AUC) calculations are in line with the ROC the results where the ultrasonic feature has the highest value with the follow-up of weighted average 5UT-1TH, difference, and average. 

### 3.2. Case 2: Debonding with Brass Inclusion—Large (12.7 mm Edge Size)

The adhesive bond containing debonding with large-brass inclusion had been inspected via ultrasonic immersion NDT and induction thermography. Feature matrices have been obtained according to the above-described (Section 2.3) post-processing methods. Data fusion algorithms (Section 2.4) have been applied to the feature matrices.

**Figure 9:**
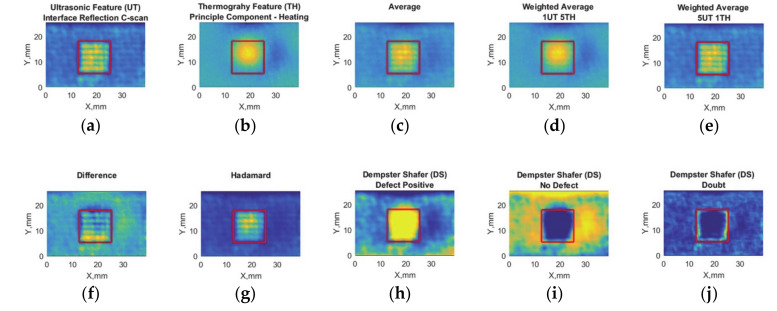
Debonding with brass inclusion (12.7 mm edge size) feature-based data fusion algorithm results: (**a**) ultrasonic feature (UT) with maximum values at interface reflection C-scan, (**b**) thermography feature (TH) principal component analysis response, (**c**) average of UT and TH, (**d**) weighted average where TH is five times more than UT, (**e**) weighted average where UT is five times more than TH, (**f**) absolute difference, (**g**) Hadamard fusion, (**h**) Dempster–Shafer fusion for defect placement, (**i**) Dempster–Shafer fusion for no defect positions, and (**j**) Dempster–Shafer fusion where doubt is high. The real position of the defect has been indicated with red squares.

The feature matrices for adhesive bond containing large-brass inclusion with ultrasonic inspection, induction thermography, and data fusion results are shown in Figure 9.

While all feature matrices indicate a contrast between defect region and sound area, Dempster–Shafer-positive (Figure 9h) and negative (Figure 9i) fusion results seem to have the highest contrast in defect detection. 

**Figure 10:**
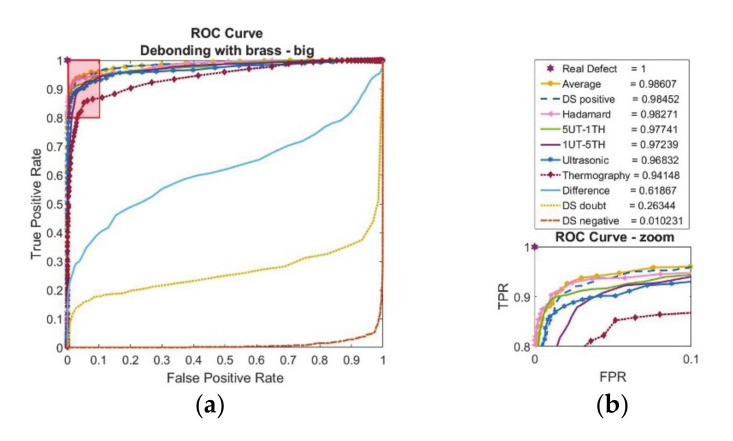
Debonding with brass inclusion (12.7 mm edge size) feature-based data fusion evaluation results with (**a**) receiver operating characteristic curve (ROC) and (**b**) area-under-curve (AUC) calculations with zoom image of ROC curve.

Quantitative evaluation results for debonding with large-brass film inclusion have been shown in Figure 10.

The performance of each technique is quite similar except the difference and DS-doubt, DS-negative. It shows that the doubt and contradiction between ultrasonic and induction thermography are very low. As seen in Figure 10b legend, the area-under-curve calculations agrees with ROC curves. While there is a small difference between each technique, basic averaging data fusion performs the best. 

### 3.3. Case 3: Debonding with Brass Inclusion—Small (6.35 mm Edge Size) 

The adhesive bond containing debonding with small-brass inclusion had been inspected via ultrasonic immersion NDT and induction thermography. Feature matrices have been obtained according to the above described (Section 2.3) post-processing methods. Data fusion algorithms (Section 2.4) have been applied to the feature matrices.

**Figure 11:**
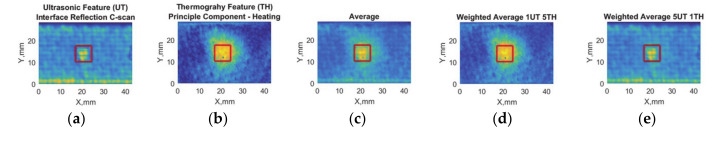

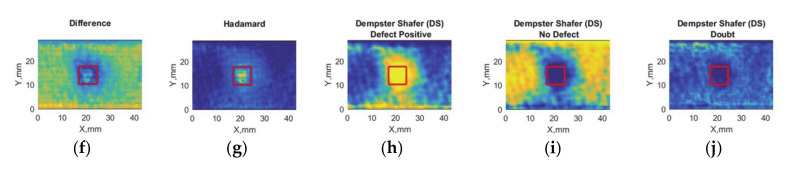
Debonding with brass inclusion (6.35 mm edge size) feature-based data fusion algorithm results: (**a**) ultrasonic feature (UT) with maximum values at interface reflection C-scan, (**b**) thermography feature (TH) principal component analysis response, (**c**) average of UT and TH, (**d**) weighted average where TH is five times more than UT, (**e**) weighted average where UT is five times more than TH, (**f**) absolute difference, (**g**) Hadamard fusion, (**h**) Dempster–Shafer fusion for defect placement, (**i**) Dempster–Shafer fusion for no defect positions, and (**j**) Dempster–Shafer fusion where doubt is high. The real position of the defect has been indicated with red squares.

Feature-based data fusion results for adhesive bonds containing debonding with small-brass inclusions are shown in Figure 11.

The results suggest ultrasonic and induction thermography NDT features can detect the defect presence, while ultrasonic feature underestimates the defect size (Figure 11a); induction thermography overestimates it (Figure 11b). The difference data fusion suggests that there is no contradiction in between NDT techniques. DS-positive seems to have the highest contrast (Figure 11h).

**Figure 12:**
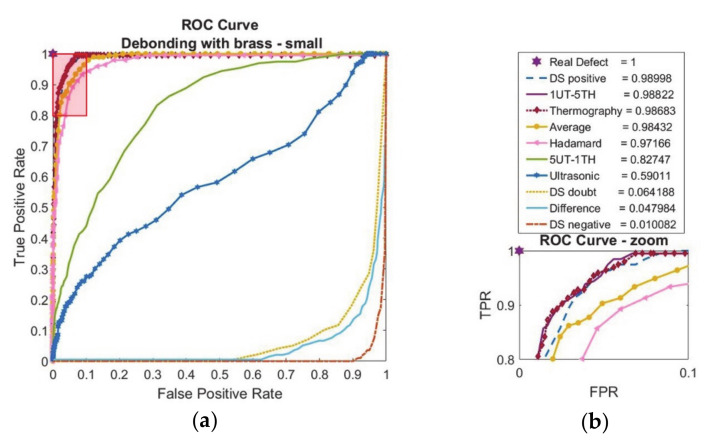
Debonding with release film brass inclusion (6.35 mm edge size) feature-based data fusion evaluation results with (**a**) receiver operating characteristic curve (ROC) and (**b**) area-under-curve (AUC) calculations with zoom image of ROC curve.

According to receiver operating curve and area-under-curve calculations for the debonding with small-brass inclusion, the ultrasonic feature and weighted average with 5UT-1TH does not perform well compared to the others (Figure 12).

While the best performance is observed at DS-positive, the induction thermography feature also performs quite well. Low AUC values for difference, DS-doubt, and DS-negative suggest that the NDT techniques do not contradict each other.

## 4. Discussion

These results indicate that data fusion algorithms can improve the debonding type defect detection performance for bonding quality evaluation. In this work, three different cases of bonding quality have been investigated by ultrasonic immersion inspection and induction thermography. The obtained data had been preprocessed for data fusion with several steps. The feature matrices that have been extracted from each nondestructive testing method results were used as the source for data fusion algorithms. The data fusion algorithms have been evaluated with quantitative sensitivity analysis.

In the case study one, a composite-adhesive single-lap joint with release film debonding artificial defect was investigated. Ultrasonic inspection with 10 MHz central frequency focused transducer detects the artificial defect fairly well (Figure 7a) due to high acoustic impedance difference between the air within the double-sided release film and single-lap joint interface. However, the induction thermography feature does not correlate well with the defect position (Figure 7b); because the electrical conductivity level of release film is quite similar to the epoxy adhesive. In this case, for induction thermography inspection, the thermal wave dominates over Joule’s effect. Therefore, the fusion algorithms that are more focused on ultrasonics, such as weighted average 5UT-1TH (Figure 7e) has higher performance than thermography dominant fusion algorithms (Figure 8). As the difference fusion matrix shows defect presence in Figure 7f and is evaluated with high values in area-under-curve calculations (Figure 8), it can be said that two NDT techniques contradict each other in case study one.

In case study two, a composite-adhesive single-lap joint containing large brass film artificial debonding was investigated. Ultrasonic immersion investigation performs well with respect to qualitative and quantitative evaluation (Figure 9a and Figure 10). However, it does not indicate clear results as good as case study one even though the defect dimensions are the same. On the other hand, compared to case study one, the high electrical conductivity of interfacial inclusion results with high temperature contrasts in thermography investigation (Figure 9b). Hence. The contradiction between two data fusion sources is much lower, as seen in the difference fusion algorithm (Figure 9e) and observed low-values for the difference in area-under curve calculations (Figure 10). While information theory-based fusion algorithms like DS perform quite well with detecting defects (Figure 9h), the basic algorithm average is evaluated better in receiver operating characteristic curve (Figure 10).

In case study three, the composite-adhesive single-lap joint with relatively small brass inclusions was investigated. Even though ultrasonic inspection results are improved by choosing a focused transducer rather than a flat transducer as in the previous work [3], the defect detection performance with ultrasonic NDT is still limited, as seen in Figure 11a and as evaluated by ROC curves (Figure 12). Since the brass inclusion has high electrical conductivity like in case two, induction thermography performed well in defect detection qualitatively, as seen in (Figure 11b) and quantitatively as calculated in the area-under-curve results (Figure 12). While the contradiction between ultrasonic NDT and thermography is low according to the difference fusion algorithm (Figure 11e and Figure 12), both basic and information theory based fusion algorithms have increased the performance of separate techniques.

When considering ultrasonic inspection of adhesively bonded structures, transducer selection plays a significant role. The small defect detection performance is increased by changing from flat transducers to focused transducers. On the other hand, in order to obtain a clear ultrasonic response in the time domain -clear from the multiple reflections within the composite-adhesive bond, a high central frequency of the transducers is required. However, due to the high frequency, the highly attenuated composite adherend causes a drastic ultrasonic amplitude decrease, which makes the defect detection challenging.

The induction thermography results show that the brass inclusions have been detected with high performance. However, the release film inclusion at the interface is not detected with the same precision as the brass inclusions. This difference in the detection performance is caused by their electrical conductivity levels. While the brass is an electrically conductive material, which allows eddy current to form within, debonding with release film only affects the thermal diffusion. Therefore, induction thermography is a successful technique to detect inclusions that are electrically conductive, even for the small sizes.

It is important to mention that both ultrasonic inspection and induction thermography have advantages and limitations for bonding quality evaluation due to their physical and practical characteristics. Although ultrasonic inspection with the immersion technique is a successful method to detect debonding with release film inclusion, it requires the samples to be underwater, which may not be applicable to every specimen. Induction thermography is, on the other hand, a noncontact NDT technique that does not require any contact medium. However, the nonconductive material inclusions and air-induced delamination may not be determined as successful as ultrasonic inspection. As these inclusions represent possible foreign object introduction to the bonding area during the manufacturing stage, both conductive and nonconductive inclusions are significant. However, at the maintenance scenario where air gap and porosity at the bondline causes debonding, only nonconductive inclusion results should be considered.

Considering three cases, it is observed that the data fusion of ultrasonic NDT with induction thermography can increase the detection performance of defect detection. While information theory-based fusion algorithms like DS perform well, the basic fusion algorithms such as Hadamard and averaging cannot be disregarded. In case study 1, ultrasonic testing performs the best; therefore, each data fusion algorithms that are favoring ultrasonic inspection, such as weighted average 5UT-1TH performs well. Also, it is seen that the area-under-curve values for the difference is close to 1, which indicated that the data fusion sources (induction thermography and ultrasonic inspection feature results) are in contradiction. In case study 2, it is observed that averaging, DS, and Hadamard improves the results from different NDT techniques. On the other hand, case 3 highlights the importance of information theory-based method DS: while averaging evaluated as lower performance than thermography, DS-positive performs very well on defect detection.

Composite-adhesive bonding nondestructive evaluation is considered one of the most challenging NDT applications. This application study only covers the detection of debonding and might not be applicable to weak and kissing bond predictions. Also, the proposed nondestructive evaluations might not suit perfectly for bonding structures with different material properties such as dissimilar joints and aluminum bonded structures. It is important to point out that the contradiction between sources and the preprocessing steps affects the performance of data fusion significantly. The limitations observed in this work might be overcome by deep learning algorithms to emphasize different features from different sources and evaluate the contradiction with statistical-based algorithms.

## 5. Conclusions

In this work, three different artificial debonding within composite-adhesive single-lap joints have been investigated with ultrasonic immersion pulse-echo technique and induction thermography. Data fusion has been used to increase the performance of different defect detection. The following points highlight the conclusions in this work.

- Ultrasonic immersion pulse-echo NDT technique is an advantageous method for debonding detection.
- Induction thermography NDT performs well with electrically conductive inclusion detection; however, it is not sensitive to nonconductive inclusions.
- While ultrasonic NDT performs better in release film inclusion, obvious fact that brass inclusion (or any inclusion with high electrical conductivity) is detected better with induction thermography.
- Data fusion performs well only if the sensors are not in contradiction.
- While the information theory-based fusion algorithm, the Dempster-Shafer rule of combination and Hadamard shows high performance, basic data fusion techniques such as averaging should not be disregarded.
- NDT of adhesive bonding is challenging, but as long as the sources do not contradict, data fusion increases the sensitivity and specificity of the inspection.

## Figures and Tables

**Table 1:** Data fusion algorithms with description and mathematical formulas.

Fusion Algorithm	Description	Mathematical Formula	
average	on pixel level, the average from two sources: UT^1^ and TH^2^	$\left(UT+TH\right)/2$	(1)
difference	on pixel level, differentiating one matrix (TH) from the other (UT)	$\left(UT-TH\right)$	(2)
Weighted average	on pixel level, weighted average when one matrix has four times higher weight than the other	$\left(\left(5xUT\right)+TH\right)/6$	(3)
		$\left(UT+\left(5xTH\right)\right)/6$	
Hadamard product	pixel-wise multiplication of same-size matrices	${\left(UT\circ TH\right)}_{ij}={\left(UT\right)}_{ij}{\left(TH\right)}_{ij}$	(4)
Dempster–Shafer rule of combination	evidence theory based on mass, belief, and plausibility functions	$\left(m_{1}⊕m_{2}\right)\left(A\right)=\frac{1}{K-1}\;\underset{B\cap C=A\neq \emptyset }{\sum }m_{1}\left(B\right)m_{2}\left(C\right)\;$ where $K=\underset{B\cap C=\emptyset }{\sum }m_{1}\left(B\right)m_{2}\left(C\right)$	(5)

^1^ UT stands for feature matrix of ultrasonic NDT. ^2^ TH stands for feature matrix of induction thermography NDT.
